# Increased levels of villus-derived exosomal miR-29a-3p in normal pregnancy than uRPL patients suppresses decidual NK cell production of interferon-γ and exerts a therapeutic effect in abortion-prone mice

**DOI:** 10.1186/s12964-024-01610-0

**Published:** 2024-04-16

**Authors:** Zheng Fang, Jiaqin Mao, Jialyu Huang, Huijun Sun, Xueyan Lu, Hui Lei, Jie Dong, Shuqiang Chen, Xiaohong Wang

**Affiliations:** 1 Center for Reproductive Medicine, Department of Gynecology and Obstetrics, Tangdu Hospital, Air Force Medical University, Xi’an, China; 2 Center for Reproductive Medicine, Jiangxi Maternal and Child Health Hospital, Nanchang, China

**Keywords:** Unexplained recurrent pregnancy loss, Decidual natural killer cell, Exosomes, Interferon-γ, miRNA

## Abstract

**Objective:**

Recurrent pregnancy loss (RPL) patients have higher absolute numbers of decidual natural killer (dNK) cells with elevated intracellular IFN-γ levels leading to a pro-inflammatory cytokine milieu, which contributes to RPL pathogenesis. The main objective of this study was twofold: first to explore the regulatory effects and mechanisms of villus-derived exosomes (vEXOs) from induced abortion patients or RPL patients at the level of intracellular IFN-γ in dNK cells; second to determine the validity of application of vEXOs in the treatment of unexplained RPL (uRPL) through in vitro experiments and mouse models.

**Methods:**

Exosomes were isolated from villus explants by ultracentrifugation, co-cultured with dNK cells, and purified by enzymatic digestion and magnetically activated cell sorting. Flow cytometry, enzyme-linked immunosorbent assays, and RT-qPCR were used to determine IFN**-**γ levels. Comparative miRNA analysis of vEXOs from induced abortion (IA) and uRPL patients was used to screen potential candidates involved in dNK regulation, which was further confirmed by luciferase reporter assays. IA-vEXOs were electroporated with therapeutic miRNAs and encapsulated in a China Food and Drug Administration (CFDA)-approved hyaluronate gel (HA-Gel), which has been used as a clinical biomaterial in cell therapy for > 30 years. In vivo tracking was performed using 1,1-dioctadecyl-3,3,3,3-tetramethylindotricarbocyaine iodide (DiR) labelling. Tail-vein and uterine horn injections were used to evaluate therapeutic effects of the engineered exosomes in an abortion-prone mouse model (CBA/J × DBA/2 J). Placental growth was evaluated based on placental weight. *IFN-γ* mRNA levels in mouse placentas were measured by RT-qPCR.

**Results:**

IFN-γ levels were significantly higher in dNK cells of uRPL patients than in IA patients. Both uRPL-vEXOs and IA-vEXOs could be efficiently internalized by dNK cells, whereas uRPL-vEXOs could not reduce the expression of IFN-γ by dNK cells as much as IA-vEXOs. Mechanistically, miR-29a-3p was delivered by vEXOs to inhibit IFN-γ production by binding to the 3′ UTR of *IFN-γ* mRNA in dNK cells. For in vivo treatment, application of the HA-Gel effectively prolonged the residence time of vEXOs in the uterine cavity via sustained release. Engineered vEXOs loaded with miR-29a-3p reduced the embryo resorption rate in RPL mice with no signs of systemic toxicity.

**Conclusion:**

Our study provides the first evidence that villi can regulate dNK cell production of IFN-γ via exosome-mediated transfer of miR-29a-3p, which deepens our understanding of maternal–fetal immune tolerance for pregnancy maintenance. Based on this, we developed a new strategy to mix engineered vEXOs with HA-Gel, which exhibited good therapeutic effects in mice with uRPL and could be used for potential clinical applications in uRPL treatment.

**Graphical Abstract:**

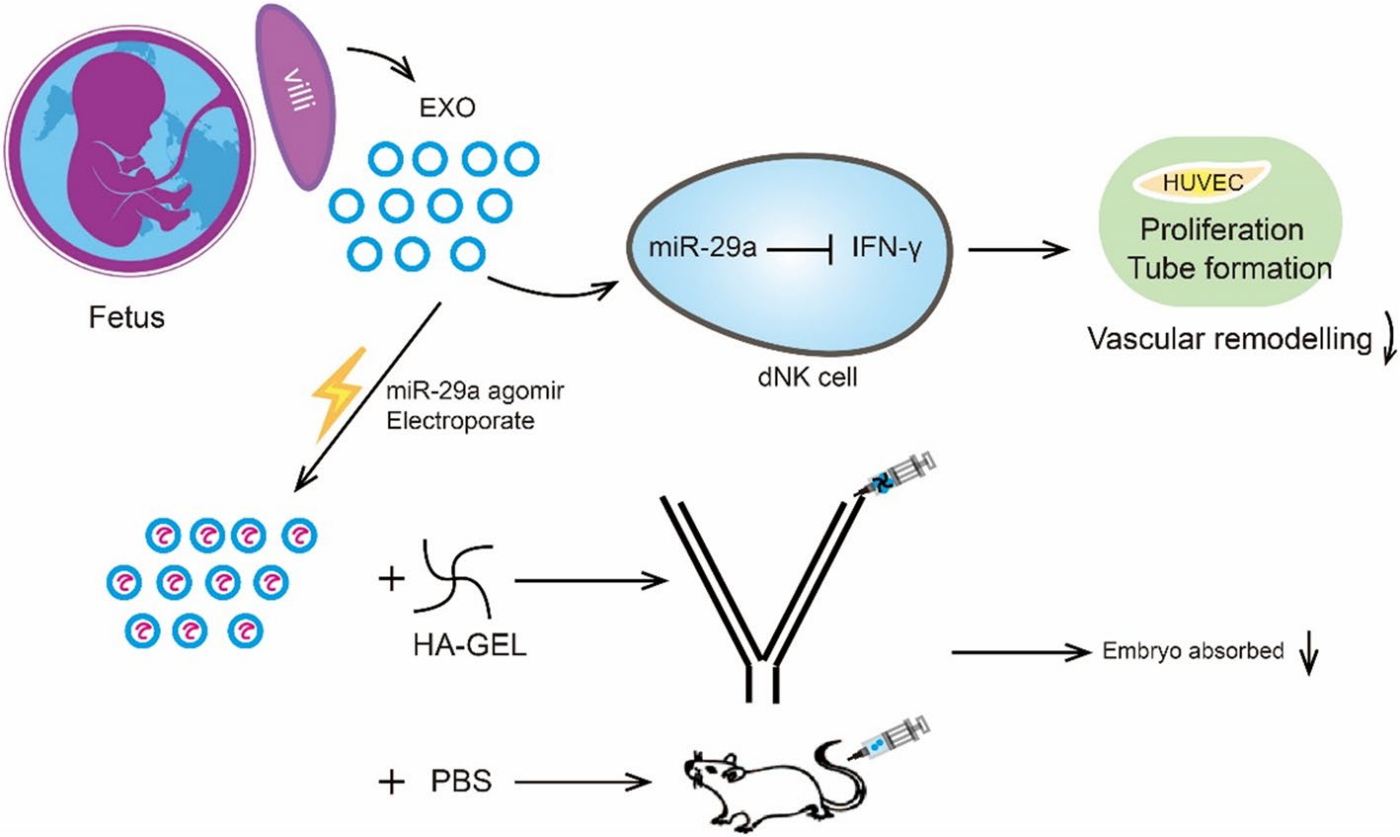

**Supplementary Information:**

The online version contains supplementary material available at 10.1186/s12964-024-01610-0.

## Introduction

RPL is a distressing pregnancy disorder affecting approximately 2.5% of women worldwide [1]. According to guidelines of the European Society of Human Reproduction and Embryology (ESHRE) and the American Society for Reproductive Medicine (ASRM), RPL is defined as two or more clinically diagnosed pregnancy losses, including embryo and fetal loss, occurring before 20–24 weeks of gestation [2]. Various mechanisms have been identified in the pathogenesis of RPL, including chromosomal abnormalities, uterine malformation, and endometrial dysfunction. Nonetheless, almost 50% of cases are uRPLs with no definitive cause, suggesting the need for further exploration and investigation [1]. Most uRPLs occur between gestational weeks 6–8, when the implantation process is completed and the maternal–fetal interface is established [3].

Pregnancy is an allogeneic semi-transplantation process; therefore, establishing appropriate maternal–fetal immune tolerance is critical [4]. As the first point of direct contact at the maternal–fetal interface, villi can induce immune tolerance in decidual immune cells by secreting various regulatory signals, such as miRNAs, proteins, and chemokines [5]. During the first trimester of pregnancy, the immune balance at the maternal–fetal interface is dominated by Th2-type cellular immunity. If the immune balance is dominated by Th1-type cellular immunity, an immune imbalance can occur and lead to various diseases such as pre-eclampsia, intrauterine growth restriction, and preterm birth [6]. More recently, studies have also revealed that approximately half of patients with uRPL demonstrate immune abnormalities at the maternal–fetal interface, especially an increase in pro-inflammatory cytokines that promotes inflammation at the maternal–fetal interface [1, 6].

In the first trimester, immune cells comprise some 40% of the total cell population in the decidua, of which 50%–70% dNK cells [7]. As the most abundant component, dNK cells play important roles in mediating immune tolerance. In contrast to the cytotoxic effects of peripheral NK (pNK) cells [8], dNK cells are CD56^bright^CD16^dim^ and appear to secrete vascular endothelial growth factor (VEGF), interferon-γ (IFN-γ), osteoglycin, pleiotrophin, and other cytokines [9, 10]. Nonetheless, in single-cell sequencing of uRPL patient decidua, it has been found that chromatin accessibility and expression levels of IFN-γ in dNK cells are significantly higher than in normal patients [11, 12], consistent with increases involving other Th- cytokines [13–18]. These findings suggest that IFN-γ derived from dNK cells may be an important regulator in the pathogenesis of uRPLs.

However, the factors that contribute to the production of elevated IFN-γ levels by dNK cells in PRL patients are not clear. Extracellular vesicles (EVs) are widely studied as mediators of intercellular communication [19]. Depending on their diameter, EVs can be divided into microvesicles, apoptotic bodies, and exosomes. Exosomes are 40–200 nm diameter vesicles with phospholipid bilayer structures containing proteins, RNAs, and metabolites [20]. It has been reported that villus-derived exosomes can regulate the induction of immune tolerance by monocytes in peripheral blood, inhibit T cell cytotoxicity, and induce T cell apoptosis [21, 22]. It has also been found that exosomes containing PD-L1 inhibit IFN-γ secretion by T cells [23]. Exosomes derived from tumor cells can inhibit NK cells [24]. However, it is unclear whether EVs derived from villi regulate IFN-γ production by dNK cells and whether dysregulation of communication between EVs and dNK cells is involved in the pathogenesis of RPL.

In this study, we assessed this question using villus-derived exosomes (vEXOs) from first-trimester villus explants of IA and uRPL patients, and dNK cells isolated from first-trimester decidua. By integrating miRNA expression profiles of vEXOs from IA and uRPL patients, we demonstrated for the first time that vEXOs represent a key regulator to down-regulate IFN-γ at the maternal–fetal interface in early pregnancy via miRNA-29a-3p. On this basis, we further developed a preclinical hyaluronate gel (HA-Gel) composite encapsulating IA-vEXOs laden with miRNA-29a-3p agomir to treat abortion-prone mice in an animal model, which could reduce the abortion rate and down-regulate IFN-γ levels in blood and placenta. Our study enhances the understanding of maternal immune tolerance to fetal allografts and provides new insights into the treatment of immune-associated uRPL.

## Materials and methods

### Human samples

Villus and decidual samples were obtained from patients with uRPL (*n* = 39) and those undergoing elective IA (*n* = 85) at the Department of Obstetrics and Gynecology of Tangdu Hospital. Donor characteristics are shown in Tables S1 and S2. The diagnosis of abortion was according to the guidelines of Tangdu Hospital, such as no fetal heartbeat for 1 week and reduced serum β-human chorionic gonadotropin and progesterone levels. Fetal cardiac activity was assessed using Doppler ultrasound at 6–8 weeks of gestation. The inclusion criteria for the uRPL group were as follows: 1) history of two or more consecutive spontaneous abortions, excluding ectopic pregnancies and molar pregnancies, including biochemical pregnancy; 2) age under 35 years and gestational age between 6–8 weeks.; and 3) normal villus chromosome karyotype. The exclusion criteria were as follows: 1) uterine structural malformations such as uterine septum, uterine myoma, and adenomyosis; 2) endocrine diseases such as diabetes, hyperprolactinemia, and hypothyroidism; 3) reproductive tract infections including chronic endometritis; 4) parental chromosomal abnormalities; and 5) a history of autoimmune diseases and thrombophilia. For the control group, age-matched patients or women with no previous pregnancy loss who underwent voluntary IA at 6–8 weeks of gestation were recruited. None of the women had a history of spontaneous abortion, pre-eclampsia, or preterm birth. The exclusion criteria were the same for patients with uRPL. All specimens were obtained after obtaining written informed consent from patients. This study protocol was approved by the Ethics Committee of Tangdu Hospital (TDLL2018-03–39).

### Preparation of exosome-depleted FBS

Exosome-depleted serum was prepared as previously described [25]. Briefly, FBS was centrifuged at 180,000 × *g* for 18 h (Optima XE-100 Ultracentrifuge, SW32Ti rotor, Beckman Coulter) and 80% of the upper layer of FBS was collected and then filtered through a 0.22 μm filter (Millipore).

### Villus explant culture

Fresh villus samples were obtained from the operating room and transported to the laboratory on ice within 10 min. Villus samples were washed in cold 1 × PBS (Corning), minced into 1 mm pieces, and transferred to Netwell™ inserts (Corning) in DMEM/F12 medium supplemented with 2% exosome-depleted FBS, 5 ng/mL epidermal growth factor (MCE), 1 × Insulin-Transferrin-Selenium Solution (MCE), 400 U/L hCG (Lizhu), 100 μg/mL streptomycin and 100 U/L penicillin (Millipore). Explants were incubated under 5% CO_2_ at 37℃ for 16 h. The same batch of FBS was used in all experiments. After incubation, the villus explants were centrifuged at 5,000 × *g* for 30 min to densify the pellets, which were then weighed after removing all traces of culture medium.

### Isolation and characterization of villus-derived exosomes

Exosomes were isolated from villus explant culture supernatants by sequential centrifugation. First, the supernatant was centrifuged (Hermle, Z446K) at 500 × *g* for 10 min to remove tissue explants, followed by centrifugation at 3,000 × *g* for 15 min and 20,000 × *g* for 2 h to remove dead cells and cell debris. The remaining supernatant was ultracentrifuged (Eppendorf, CP80NX) at 120,000 × *g* for 90 min and the pellet was washed with cold PBS and ultracentrifuged again. The final exosome pellets were resuspended in 100 μL of PBS and stored in low protein-binding Eppendorf tubes at 2–8℃ for 1 week and at -80℃ for long-term storage. All of the steps above were performed at 4℃ in a laboratory environment.

Nanoflow cytometry was used for size distribution analysis and exosome morphology was determined using transmission electron microscopy (TEM). These two procedures were performed by LC-Bio-Technologies (Hangzhou) Co., Ltd., as previously reported [26]. Exosomal markers were identified by immunoblotting, as previously described [27]. Exosome surface markers were detected by immunoblotting using anti-CD9 (ab263019, 1:1,000; Abcam), anti-CD63 (ab134045, 1:2,000; Abcam), anti-calnexin (ab133615, 1:2,000; Abcam), and anti-HLA-G (ab52455, 1:2,000; Abcam) antibodies. To measure the protein levels of exosomes, a bicinchoninic acid (BCA) kit (Thermo Scientific) was used according to the manufacturer’s instructions.

### Isolation of human dNK cells

Fresh decidual samples were obtained from the operating room and transported to the laboratory on ice within 10 min. Decidual samples were washed with cold 1 × PBS (Corning) and minced into 1 mm pieces. The tissue pieces were then transferred to a 50 mL centrifuge tube (Corning) and digested with 1 mg/mL collagenase type IV (Sigma-Aldrich) and 0.025 mg/mL DNase I (Sigma-Aldrich) in RPMI 1640 medium (Gibco) for 1 h in a 37℃ water bath with continuous shaking. The suspensions were strained through 100 μm, 70 μm, and 40 μm nylon mesh (Falcon) and centrifuged at 220 × *g* for 10 min. Erythrocyte lysis buffer (Miltenyi Biotec) was used to remove erythrocytes from the collected cells. The collected cells were washed twice with RPMI-1640 and resuspended in 50 mL RPMI-1640 medium containing 10% FBS (Corning), 1 × non-essential amino acids, 1 × GlutaMAX and 1% penicillin–streptomycin (all from Gibco). The cells were cultured in T75 cell culture flasks for 2 h at 37℃ in a humidified 5% CO_2_ incubator (Thermo) to remove decidual stromal cells and macrophages. Only non-adherent cells, also called decidual immune cells, were collected and washed twice with complete medium. NK cells were further purified by negative selection using a magnetic-activated cell sorter (Miltenyi Biotec). The purity of the resulting dNK cells was > 94% CD56^+^. To simulate the in vivo state of dNK cells in patients, 0.05 ng/mL recombinant human IL-12 protein (R&D Systems, 10,018-IL) was added to the medium.

### Exosome labelling with PKH26

Exosomes (500 μg) were incubated with 100 μL PKH26 working solution (Sigma) at 25℃ for 10 min away from light. After incubation, 5 mL of 1 × PBS was added to terminate labeling, and the exosome isolation protocol was repeated to remove free dyes.

### Co-culture of exosomes and dNK cells

For incubation with primary dNK cells, purified exosomes were resuspended in complete RPMI-1640 medium to a final concentration of 100 μg/mL. The same volume of PBS was added to the medium as a negative control. The mixtures were exposed to purified dNK cells and incubated for 12 h or longer.

### Enzyme-linked immunosorbent assay (ELISA)

Concentrations of IFN-γ in dNK cell supernatants or tissue protein extracts were quantified using a human IFN-γ ELISA kit (R&D Systems), which was used according to the manufacturer’s instructions.

### RNA isolation and RT-qPCR

Long RNAs (> 200 bp) were extracted from purified human decidual NK cells treated with different interventions, using an RNA purification kit (Thermo Scientific). We extracted miRNAs from decidual immune cells and exosomes using RNAiso as a small RNA reagent (TaKaRa). Reverse transcription of long RNAs was performed using PrimeScript™ RT Master Mix (TaKaRa) and Mir-X™ miRNA FirstStrand Synthesis reagent (TaKaRa) was used for miRNA. miRNA expression levels were normalized to the external reference gene cel-miR-39 (Qiagen), a nematode miRNA that is not expressed in humans or mice [28]. The resulting cDNA was analyzed for expressed genes by real-time quantitative RT-qPCR with the One Step TB Green PrimeScript RT-PCR Kit II (TaKaRa Bio) or TB Green Advantage qPCR Premix (TaKaRa Bio). PCR primers used in experiments here are listed in Table S5.

### Flow cytometry

Flow cytometry was performed according to a protocol provided by Biolegend.

### Tube formation assay

Tube formation assays were performed as described previously [29]. Briefly, conditioned media were collected from treated dNK cells supernatants and centrifuged at 20,000 × *g*, 37℃ for 2 h. Then, 1.2 × 10^5^ of human umbilical vein endothelial cells (HUVECs) were resuspended in different dNK cell-conditioned media, seeded into 24-well plates pre-coated with 300 μL Matrigel (R&D Systems), and incubated at 37℃ for 6 h. Finally, the cells were photographed using a 10 × brightfield objective lens (Olympus IX73). At least three different fields of view were captured per well, and the experiment was independently repeated three times.

### Exosomes electroporated with miRNA

Exosome electroporation was performed according to a protocol provided by Bio-Rad. A mixture of 0.5 OD miRNA (cel-miR-39, miR-29a-agomir/antagomir, GenePharma) and 200 μg exosomes were transferred to a 0.2 cm-gap electroporation cuvette and placed on ice. The electroporation system was set at 350 V, 150 mF, with two pulses. After electroporation, the samples were placed on ice for 30 min, incubated with RNase to remove non-loaded miRNAs attached to the exosome surface, and used for subsequent experiments.

### Development and characterization of exosomes mixed with HA-Gel

Modified exosomes were added to commoditized HA-Gel (5 mg/mL; Bioregen, Co., Ltd., China, approved by the CFDA as a medical device [No. 20153641542]) at a ratio of 3:7 (w/w). To evaluate viscosity and injectability, we extruded the gel slowly through a 29G syringe needle, and a DHR-2 (TA Instruments) rheometer was used to evaluate rheological behavior. Furthermore, vEXOs-HA-Gel was incubated at 37 °C under a 5% CO_2_ atmosphere and weighed for seven days. Saturated rhodamine B (Mackun) solution was mixed with HA-Gel to the same concentration as modified exosomes and then the mixture was incubated in PBS (Hyclone) at 37 °C in a 5% CO_2_ atmosphere. The supernatant was removed and fresh PBS was added daily. The concentration of rhodamine B released into the PBS was then determined by measuring the optical absorbance at 554 nm using a full-wavelength microplate reader (Molecular Science).

### Exosome labelling with DiR and in vivo tracking

Exosomes (approximately 2 μg/μL at the protein level) were incubated with 100 μM DiR (Umibio) at a ratio of 10:1 (v/v) at 37℃ for 30 min. After incubation, 10 mL of 1 × PBS was added to terminate labelling, and the exosome isolation protocol was repeated to remove free dyes.

Considering that mice gradually gain weight during pregnancy, each mouse was tail-vein injected with approximately 5 μg of DiR-labeled exosomes per gram of body weight according to the indicated treatment plan. The distribution of DiR-labeled exosomes in the whole body and major organs of the mice was detected using an IVIS Lumina II in vivo imaging system (Xenogen) after injection or uterine horn injection.

### dNK cells transfected with miRNA

Lipofectamine™ RNAiMAX transfection reagent (Invitrogen) was used to transfect miRNA into dNK cells according to the reverse transfection protocol provided by Invitrogen. Briefly, an miRNA oligo or transfection reagent was diluted in Opti-MEM I Reduced Serum Medium (Gibco) and incubated at room temperature for 5 min to prepare RNAi duplex-Lipofectamine RNAiMAX complexes. The complexes were added to each well with an appropriate number of dNK cells and incubated for 24 h at 37℃ in a CO_2_ incubator. After incubation, the dNK cells were collected for RNA extraction and flow cytometry. The RNA oligo sequences used in these experiments are listed in Table S5.

### Luciferase reporter-gene assay

The full-length wild-type or mutant 3′ UTR of *IFN-γ* was cloned into firefly luciferase reporter plasmids (Promega). HEK293T cells were co-transfected with a mixture of firefly luciferase reporter plasmids, pRL-TK Renilla luciferase plasmid (Promega), and appropriate constructs or miRNAs (GenePharma). Luciferase activity was measured using the Dual-Luciferase Reporter Assay System (Promega) as previously described [30]. Transfection efficiency data were normalized to the ratio of firefly luciferase to Renilla luciferase activity. TargetScan was used to predict the binding site of miRNAs and 3′ UTR of *IFN-γ* according to a previous description [31].

### In vivosafety evaluation

To investigate the safety of agomir-IA-vEXOs, major organ tissues were collected for histochemical analysis 24 h after the last administration. Organs were fixed in 4% paraformaldehyde for 48 h and embedded in paraffin. Each section was sectioned into 5 mm slices, processed for routine hematoxylin and eosin (H&E) staining, and visualized under a microscope (Nikon Ti).

### Animals studies

Wild-type mice (BALB/c, DBA/2 J males, and CBA/J females) were purchased from Beijing Huafukang Biotechnology Co., Ltd., China. All mice were housed in a standard specific pathogen-free laboratory with free access to food and water, controlled temperature of 24℃ to 26℃, a humidity of 65%-70% and a simulated natural lighting cycle of 12 h from 07:00 to 19:00. All experimental procedures involving animals were conducted in accordance with the National Guidelines for Animal Usage in Research (China). Permission for animal studies was obtained from the Ethics Committee of Tangdu Hospital.

To construct the RPL mouse model and healthy control model, virgin female CBA/J mice were randomly introduced into DBA/2 J or Balb/c male mice, and the timing of conception was determined by detection of a vaginal plug as gestation days (GD) 0.5.

Uterine horn injection: The mice were operated on through the back approach, the uterine horn was gently pulled out, and the skin was sutured after slowly injecting liquid with a syringe. Mice were placed on a heat preservation board for 2 h and then returned to their cages. The mated mice were injected with 50 μL HA-Gel or PBS containing exosomes at GD 4.5 and GD 7.5. Sham and RPL groups were operated on and injected with PBS to exclude any influence of the operation.

Placental processing: Placental tissues were collected 24 h after the last administration, and each placenta was drained of surface liquid using absorbent paper and weighed. The placentas were randomly selected and used for subsequent analyses.

### Statistics

Statistical analyses are summarized in the Figure legends. In most cases, the results represent means ± standard error of the mean (SEM) of n separate experiments. Two-tailed Student’s *t*-tests were used to compare two groups. Statistical significance was set at *P* < 0.05. One-way analysis of variance (ANOVA) was performed to determine whether there was a statistically significant difference between more than two datasets, followed by Bonferroni’s post-hoc correction.

## Results

### Characterization of villus-derived exosomes

**Fig. 1:**
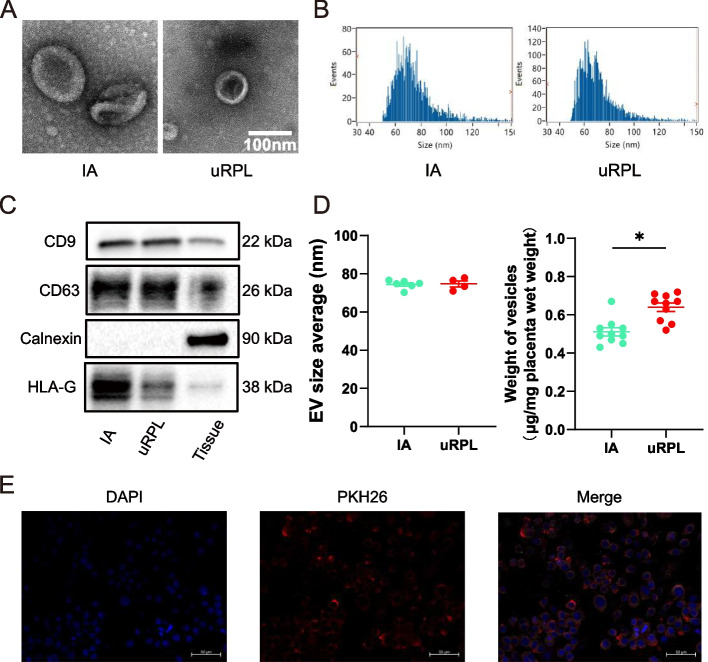
Extracellular vesicles isolated from villi explants can be absorbed by dNK cells. **A** Representative transmission electron microscopy (TEM) image of two groups of EVs. Bar, 100 nm. **B** EVs size was measured by nano-flow cytometry. **C** Western blot analysis of EVs markers, CD9, CD63, Calnexin and HLA-G were analyzed. Tissue stands for villous tissue. **D** Size distribution (left) and weight of vesicles produced (right) as determined by nano-flow cytometry and BCA kit. **E** Fluorescence microscopy images of dNK cells incubated with PKH26-labeled IA-vEXOs (red) for 24 h (scale bar, 50 μm). Nuclei were stained blue with DAPI. IA-vEXOs were stained red by PKH26. Bar, 20 μm. Results are the mean ± SEM (*n* = 4–10). ^*^ *P* < 0.05 vs. IA. Two-tailed t test (**D**)

The morphology and size of villus exosomes were assessed by TEM and flow cytometry. Specifically, exosomes from both IA and uRPL villi had typical cup-shaped bilayer membrane structures (Fig. 1A). Nanoflow cytometry showed that the exosome particle size was distributed between 40 and 150 nm, with an average of 70–75 nm (Fig. 1B). As shown in Fig. 1C, the selected molecular expression was in good agreement with the MISEV2018 guidelines [32]. In addition, we found no significant difference in the average particle diameter of exosomes between the two groups; however, uRPL-villi produced more exosomes per gram of villus weight compared with IA villi, as measured by the total protein amount (Fig. 1D).

### Villus-derived exosomes of IA patients have stronger function to downregulate IFN-γ expression by dNK cells in vitro than uRPL-EXOs

**Fig. 2:**
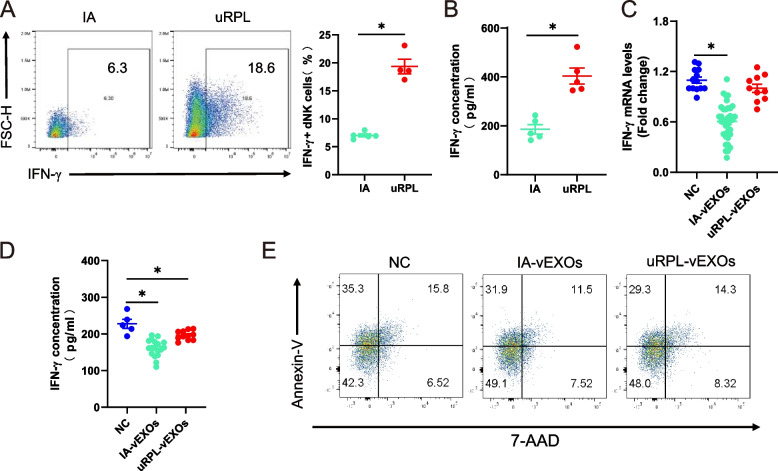
IFN-γ is overexpressed in uRPL and villi-derived exosomes downregulate IFN-γ expression of dNK cells in vitro. **A** IFN-γ + dNK cells population as revealed by flow cytometry analysis. Statistical data shown in the right panel. **B** ELISA assay for IFN-γ concentration in decidual tissue. **C** qRT-PCR analysis for IFN-γ expression level in dNK cells cultured with PBS(*n* = 14), IA-EVs(*n* = 36) or uRPL-EVs(*n* = 10). **D** ELISA assay for IFN-γ concentration in supernatant of dNK cells cultured with PBS(*n* = 5), IA-EVs(*n* = 18) or uRPL-EVs(*n* = 10). **E** Annexin V apoptosis assay for dNK cells following a 24 h co-culture with EVs. ^*^ *P* < 0.05 vs. IA (**A**, **B**) or NC (**C**). Two-tailed t test (**A**, **B**) and ordinary one-way ANOVA (**C**, **D**)

Single-cell sequencing of uRPL decidua revealed abnormally elevated IFN-γ levels in dNK cells [12]. Consistently, we found that IFN-γ was significantly overexpressed in decidua and dNK cells of uRPL patients compared with IA women (Fig. 2A, B). To further investigate the regulatory effect of vEXOs on IFN-γ in dNK cells, we co-cultured uRPL and IA vEXOs with primary dNK cells of > 90% purity. PKH26 labeling showed that exosomes were efficiently internalized by dNK cells (Fig. 1E). IA-vEXOs significantly reduced the expression of IFN-γ in dNK cells, whereas uRPL-vEXOs failed to reduce the expression of IFN-γ to the same the degree as IA-vEXOs (Fig. 2C, D). Studies have shown that extra-villus trophoblasts (villus explants) promote immune cell apoptosis [33]. However, our results failed to produce the same results, which therefore confirmed that the downregulation of IFN-γ is due to the reduced expression of IFN-γ by exosomes rather than apoptosis or death of dNK cells (Fig. 2E).

### Villus-derived exosomal miRNAs modify IFN-γ expression by dNK cells

**Fig. 3:**
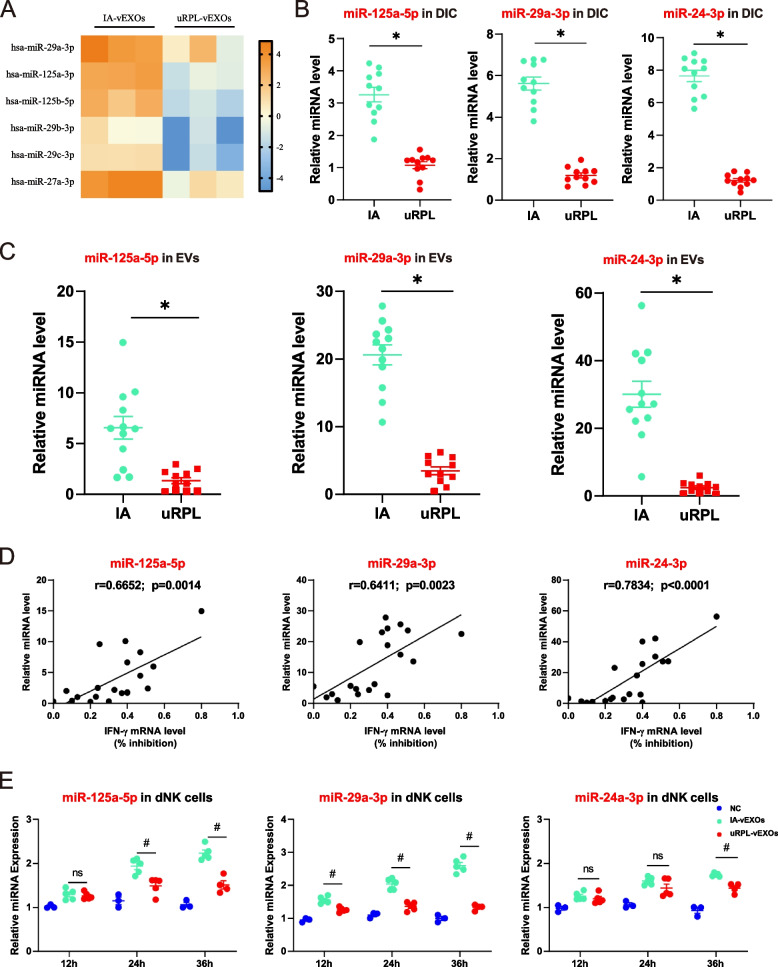
IA-EVs contain IFN-γ modifying miRNAs that are taken up by dNK cells. **A** Heatmap view of the selected miRNA candidates in the IA-vEXOs and uRPL-vEXOs from miRNAomic sequence raw data. **B** Levels of miR-24-3p, miR-29a-3p and miR-125a-5p in decidual tissues obtained from the IA or of patients with uRPL as indicated. *n* = 10 biological replicates of a representative experiment. **C** Levels of miR-24-3p, miR-29a-3p and miR-125a-5p in EVs isolated from the IA or of patients with uRPL as indicated. RNA was extracted from 100 μg of EVs isolated from each patient. The expression levels of the indicated miRNA species were measured by qRT-PCR and normalized to the levels of a spike-in control (cel-miR-39). *n* = 10 biological replicates of a representative experiment. **D** Spearman’s correlations between the relative miRNA levels of miR-24-3p, miR-29a-3p and miR-125a-5p in individual patient’s EVs and their respective effect on the inhibition ability on the production of IFN-γ of dNK cells. These include patients shown in Fig. 3B-E summarized in Table S4. **E** Levels of miR-24-3p, miR-29a-3p and miR-125a-5p in dNK cells after treatment with EVs. *n* = 10 biological replicates of a representative experiment. ^*^ *P* < 0.05 vs. IA (**B**, **C**) or NC (**E**). ^#^ *P* < 0.05 vs. IA-vEXOs. Two-tailed t test (**B**, **C**), two tailed spearman’s correlation analysis (**D**) and ordinary one-way ANOVA (E).

Exosomes have been reported to deliver different cargoes that exert immunomodulatory functions [34]. To further characterize the miRNA cargos of vEXOs, we performed miRNA sequencing of three independent samples of patients with IA and uRPL matched for age, BMI, and gestational age (Table S3). We intersected miRNAs that were highly expressed in vEXOs (High: The number of reads in the following reported miRNAs = higher than the average copy number of the data set after normalization), differentially expressed between two groups, and predicted by TargetScan (www.targetscan.org) to be able to bind to IFN-γ (Fig. 3A). Based on these standards, three miRNAs were finally selected as candidates and confirmed by RT-qPCR to be significantly downregulated in uRPL decidual immune cells (DICs) and vEXOs compared to the IA group (Fig. 3B, C). To investigate their potential role in IFN-γ regulation, individual vEXOs were collected from 20 patients (Tab. S4) and IFN-γ expression levels were measured in co-treated dNK cells. As shown in Fig. 3C, levels of the three miRNAs in vEXOs were significantly correlated with their inhibitory effect in terms of IFN-γ expression, suggesting an immune modulatory ability in fetal-maternal communication (Fig. 3D). Consistently, exposure of dNK cells to uRPL-vEXOs resulted in significantly lower miRNA levels than exposure to IA-vEXOs at different time points, suggesting that vEXOs could not only carry but also transfer these three miRNAs into dNK cells (Fig. 3E).

### miR-29a-3p agomir inhibits IFN-γ production in primary IA dNK cells

**Fig. 4:**
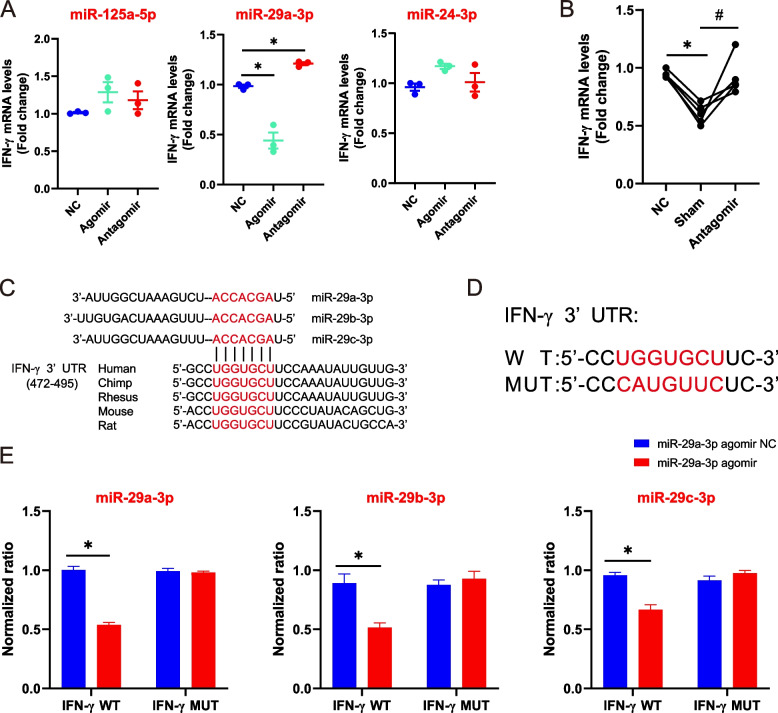
Inhibition of IFN-γ production by miR-29a-3p in primary dNK cells. **A** Effect of miRNA agomirs and antagomirs on IFN-γ production. dNK cells were transfected with 100 nM of miR-24-3p, miR-29a-3p miR-125a-5p and a control sequence (with no known target). *n* = 3 biological replicates of a representative experiment. **B** Knockdown of miR-29a-3p in EVs reverse the attenuating effects on IFN-γ production. *n* = 5 biological replicates of a representative experiment. **C** Diagram depicted the predicted binding sites of miR-29 family on the 3’-UTR of IFN-γ in different species. **D** Wild and mutant sequences of the binding site of miR-29a-3p to IFN-γ. **E** Dual luciferase reporter gene assay for verifying the direct binding site of miR-29 family and IFN-γ. *n* = 10 biological replicates of a representative experiment. ^*^ *P* < 0.05 vs. NC (**A**) or RNA oligo agomir NC (**E**). ^#^ *P* < 0.05 vs. Sham (**B**). Ordinary one-way ANOVA (**A**), RM one-way ANOVA (**B**) and two-tailed t test (**E**).

To verify the regulatory function of miRNAs in IFN-γ production, we transfected primary dNK cells with agomir and antagomir of miR-24-3p, miR-29a-3p, and miR-125a-5p or a nonsense control sequence. Considering that primary dNK cells are difficult to transfect, we confirmed transfection efficiency using RT-qPCR and FAM-labeled control transfection (Fig S1A, B). Nonetheless, only transfection with miR-29a-3p agomir inhibited the expression of IFN-γ while miR-24-3p and miR-125a-5p did not influence IFN-γ expression levels (Fig. 4A). Conversely, miR-29a-3p antagomir (chemically modified, single-stranded antisense RNA molecule) transfection significantly increased IFN-γ expression. These results indicated miR-29a-3p as a strong candidate to modulate IFN-γ expression levels.

To confirm that miR-29a-3p mediates the attenuating effect of vEXOs on IFN-γ expression, we loaded miR-29a-3p antagomir into IA-vEXOs via electroporation. To verify electroporation efficiency, we used cel-miR-39 as an indicator, which is non-homologous between humans and mice, and TEM showed that morphology of the exosomes did not change significantly after electroporation (Fig S1C, D). Consistently, inhibition of miR-29a-3p in IA-vEXO effectively reversed the reduction in IFN-γ expression levels in primary dNK cells induced by IA-vEXOs (Fig. 4B).

Based on the TargetScan database of predicted miR-29 family members and the 3′ UTR sequence of *IFN-γ* mRNA, we found that the binding sites were conserved across humans and mice (Fig. 3C). To confirm the predicted binding sites, we used luciferase reporter-gene assays, which showed that the miR-29 family significantly decreased luciferase activity of the wild-type (WT) *IFN-γ* 3’-UTR construct, but did not affect luciferase activity of the mutated *IFN-γ* 3’-UTR construct, suggesting direct regulation of miR-29 family members on IFN-γ expression (Fig. 3D, E).

Taken together, these data suggest that vEXOs could deliver miR-29a-3p to modulate IFN-γ expression by dNK cells.

### Exosomes encapsulating miR-29a-3p agomir downregulate uRPL dNK IFN-γ expression in vitro

**Fig. 5:**
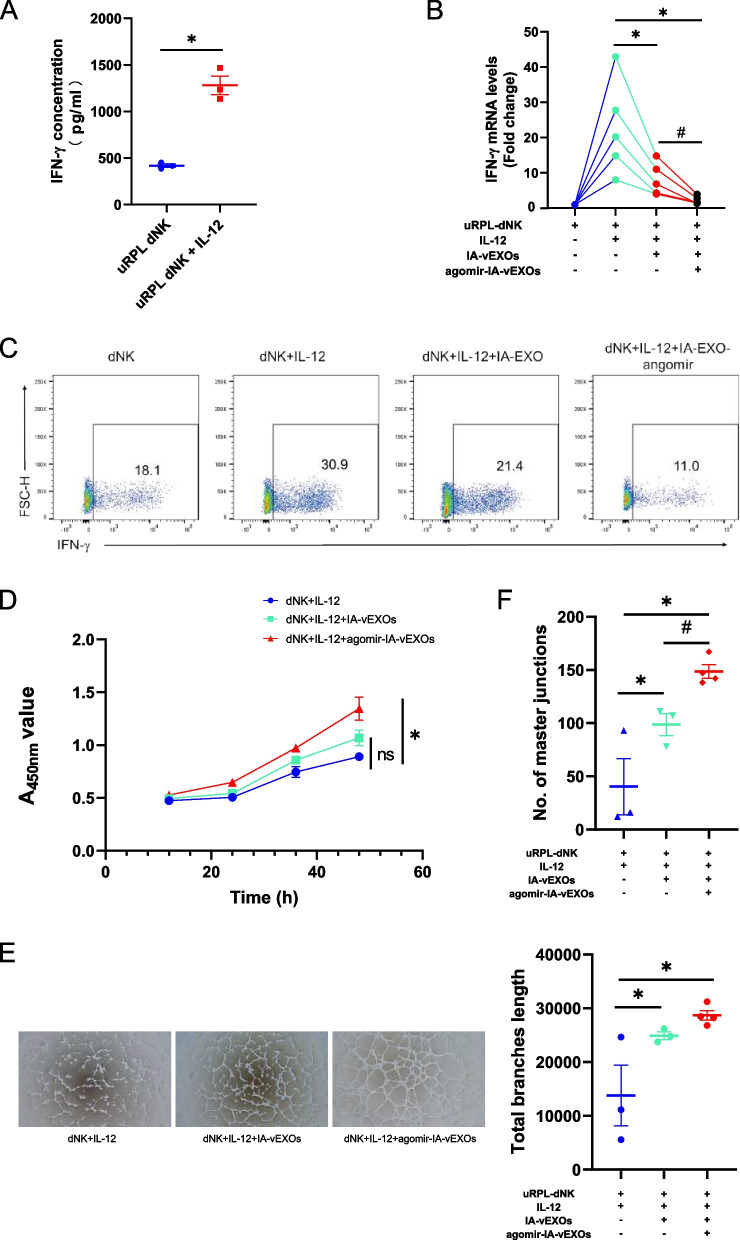
Exosomes encapsulating miR-29a-3p agomir downregulate uRPL dNK IFN-γ expression in vitro. **A** ELISA assays identify the concentration of IFN-γ in dNK cells supernatant after stimulation of IL-12. **B** qRT-PCR assay demonstrated the effect of IA-vEXOs and agomir-IA-vEXOs on IFN-γ production. **C** Representative flow cytometry images of the expression level of IFN-γ in dNK cells after treatment with IA-vEXOs and agomir-IA-vEXOs. **D** Effect of dNK cells supernatant on proliferation capacities of Huvecs by CCK-8 assay. **E** Tube formation assay demonstrated the effect of dNK cells supernatant on Huvecs. **F** Number of master junctions and total branches length analysis of (**E**). ^*^ *P* < 0.05 vs. uRPL dNK (**A**) or uRPL-dNK + IL-12 (**B**, **D**). ^#^ *P* < 0.05 vs. dNK + IL-12 + IA-vEXOs (**B**, **F**). Two-tailed t test (**A**) and ordinary one-way ANOVA (**B**, **D**, **F**)

We then investigated whether IA-vEXOs could be harnessed to deliver nucleic acid drugs for targeted therapy of high IFN-γ levels in uRPL. Previous literature reports that IL-12 is highly expressed in serum and decidua of uRPL patients [13], and IL-12 can directly stimulate IFN-γ production by NK cells [35]. Therefore, we stimulated uRPL dNK cells with recombinant IL-12 to mimic the in vivo environment. As shown in Fig. 5A, uRPL dNK cells up-regulated the expression of IFN-γ after stimulation with IL-12. We then loaded IA-vEXOs with miR-29a-3p agomir and co-cultured the exosomes with uRPL dNK cells to test the effects of agomir-IA-vEXOs on IFN-γ expression. Treatment of uRPL dNK cells with IA-vEXOs and agomir-IA-vEXOs significantly reduced mRNA levels of *IFN-γ* and agomir-IA-vEXOs exhibited a significantly stronger inhibitory ability (Fig. 5B). Furthermore, we performed flow cytometry to determine IFN-γ expression, which was consistent with cognate mRNA levels (Fig. 5C). Given the role of IFN-γ in vessel stability, we tested whether supernatants of the above dNK cells could influence proliferation and tube formation by HUVECs. As shown in Fig. 5D-F, agomir-IA-vEXOs effectively rescued the impairment of HUVEC proliferation and tube formation induced by IL-12 co-treatment, the degree of which was significantly greater than that of IA-vEXOs.

### Characterization of HA-Gel-laden exosomes

**Fig. 6:**
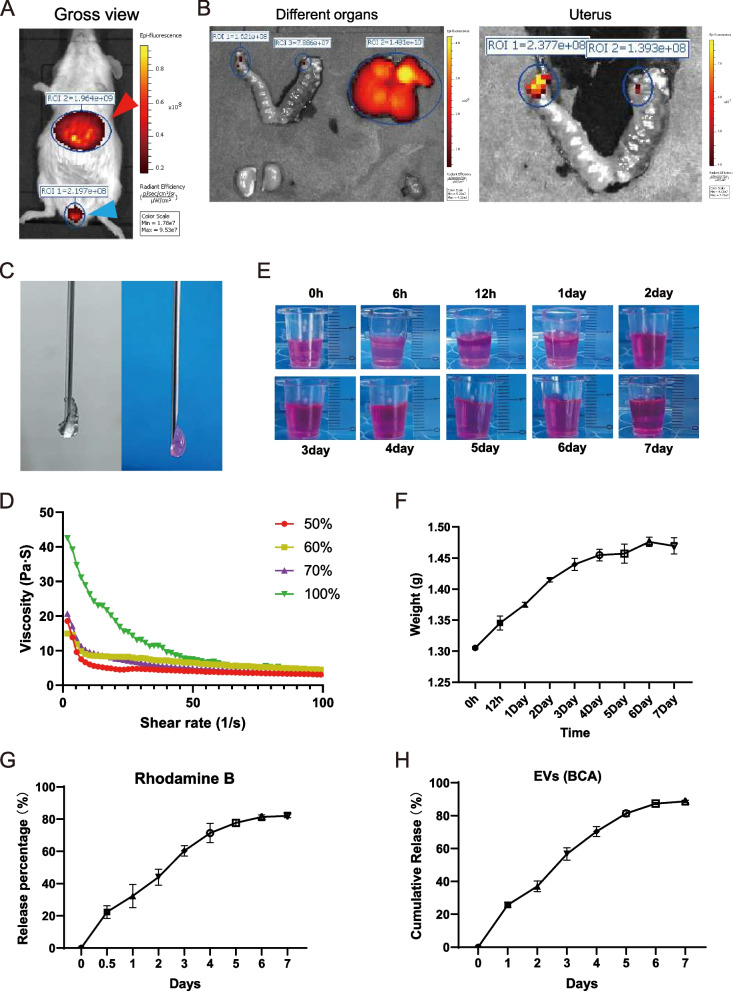
PBS is not suitable for exosomes intrauterine injection and exosomes laden HA-GEL characterization. **A**, **B** Near-IR fluorescence images of the body and main organs after tail vein injection 6 h and intrauterine injection 1 h. The red arrow represents the liver and the blue arrow represents the vaginal orifice. **C** General appearance of original HA-Gel (left) and 70% HA-Gel mixed with 30% saturated PBS solution with Rhodamine B (right). **D** Viscosity and shear rate of different concentration of HA-Gel. **E** Images of vEXOs-HA-Gel degradation in PBS solution (left). vEXOs-HA-Gel were loaded in a transwell chamber and entirely placed in PBS solution at 37℃. **F** Weight of vEXOs-HA-Gel degradation in PBS solution. **G**, **H** The curves of vEXOs-HA-Gel sustained release ability of Rhodamine B and exosomes. Rhodamine B was detected by absorbance, and exosome release was quantified by BCA assay

Next, we explored the therapeutic effects of exosomes engineered with the miR-29a-3p agomir in mouse RPL models. To better mimic clinical applications, we prioritized the intrauterine injection of exosomes. We used 1,1'-dioctadecyl-3,3,3',3'-tetramethylindotricarbocyanine iodide (DiR) labelled cel-mir-39-IA-vEXOs to acquire near-IR fluorescent images from the body and main organs via an IVIS Spectrum System. Similar to most exosome administration methods, we initially used PBS as a carrier for exosomes for intrauterine injections [36]. Tail vein-injected exosomes were abundantly enriched in the liver after 6 h, while exosomes injected through the uterine horn accumulated in the ostium vaginae and could not be detected in the uterus. By imaging the uterus alone, we found that there was only one fluorescent signal at the two injection sites, indicating that most exosomes were excreted (Fig. 6B).

Thus, we mixed exosomes with HA-Gel (5 mg/mL; Bioregen, Co., Ltd., China, approved by the CFDA as a medical device [No. 20153641542]) at a ratio of 3:7 for intrauterine therapy. HA-Gel and vEXOs-HA-Gel existed in a non-liquid form, and their viscosities for injection were evaluated using a 29G needle (Fig. 6C) and a rheometer (Fig. 6D). We also evaluated the degradation rate of vEXOs-HA-Gel in a Transwell insert surrounded by PBS solution, which showed shape maintenance for 7 days with a slight mass increase (Fig. 6E, F). Simultaneously, we determined the sustained release ability of vEXOs-HA-Gel by detecting the absorbance of rhodamine B and protein concentrations in the upper PBS solution (Fig. 6G, H).

### Agomir-IA-vEXOs alleviate pregnancy loss by downregulating IFN-γ in vivo

**Fig. 7:**
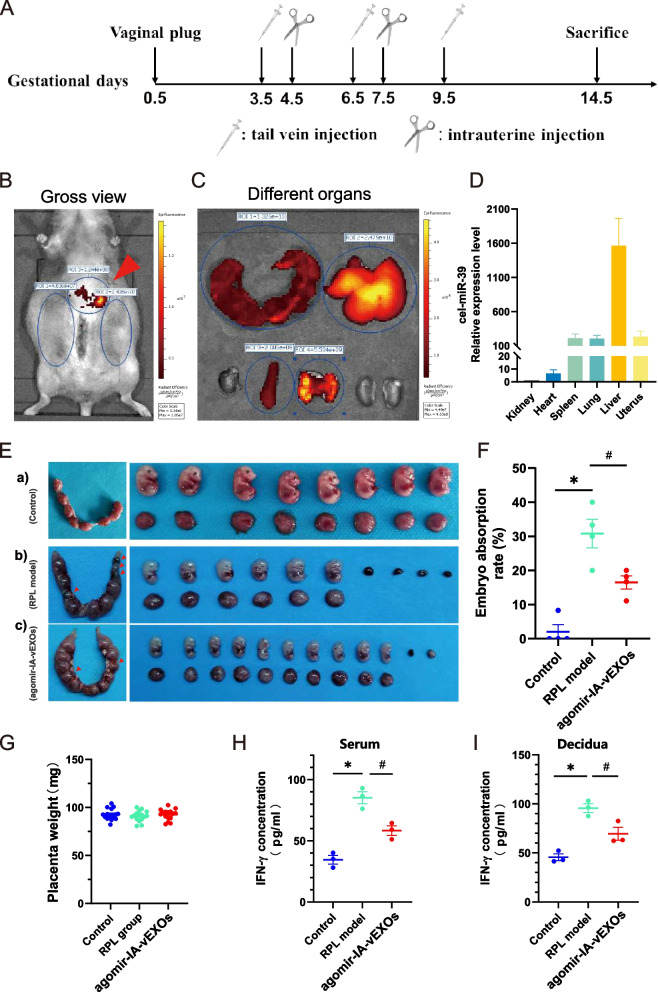
Agomir-IA-vEXOs alleviate pregnancy loss by downregulating IFN-γ in vivo. **A** Schematic diagram of the experimental process. Pregnant mice were tail vein injected with pooled agomir-IA-vEXOs from IA patients (about 100 μg (at protein level) in 100 μL) on GD 3.5, GD 6.5 and GD 9.5; and intrauterine injected with vEXOs-HA-Gel containing about 100 μg exosomes on GD 4.5 and GD 7.5. Mice were sacrificed at GD 14.5 for analysis. **B** Near-IR fluorescence images of a GD14.5 body. **C** Near-IR fluorescence images of main organs. **D** Cel-miR-39 expression level in indicated tissues after the mice received tail vein injection and intrauterine injection. Data are expressed as mean ± SEM of at least 3 biological replicates. **E** Representative uterus general image of control, RPL model and agomir-IA-vEXOs treated groups (left), red arrow represents the abortion point. The above row shows the fetus, and the bottom row shows the placenta (right). **F** Statistical data of the embryo absorption rate in each group (*n* = 4). **G** Statistical data of the placenta weight in each group (*n* = 16). (H-I) ELISA assay determined the treatment effect on the IFN-γ concentration of serum and decidual of GD 14.5 mice (*n* = 4). ^*^ *P* < 0.05 vs. Control (F, H, I). ^#^ *P* < 0.05 vs. RPL model (F, H, I). Ordinary one-way ANOVA (F-I)

To further evaluate the in vivo effects of agomir-IA-vEXOs, pregnant mice were subjected to tail vein injections of agomir-IA-vEXOs and intrauterine injections of agomir-IA-vEXOs-HA-Gel, as previously described [37] (Fig. 7A). At GD 14.5, the distribution of cel-mir-39-vEXOs was observed, with fluorescent signals in the uterus, liver, heart, spleen, lungs, and kidneys (Fig. 7B, C). Consistently, quantitative polymerase chain reaction (qPCR) analysis revealed that cel-miR-39 mainly accumulated in the liver, lung, uterus, and spleen (Fig. 7D), implying the in vivo absorption of vEXOs.

The RPL mouse and healthy control models were constructed by mating virgin CBA/J female mice with DBA/2 J and Balb/c male mice, followed by a sham operation for the agomir-IA-vEXO treatment group. The embryo resorption rate in the RPL model was 20–40% and that of the control group was 11.1–20%, whereas placental weights showed no significant differences (Fig. 7E-G). As previously reported [38], the RPL model showed a higher IFN-γ level in peripheral blood and decidua. In contrast, the administration of agomir-IA-vEXOs significantly decreased the embryo resorption rate as well as IFN-γ concentrations (Fig. 7H-I).

### Safety evaluation of agomir-IA-vEXOs treatment

**Fig. 8:**
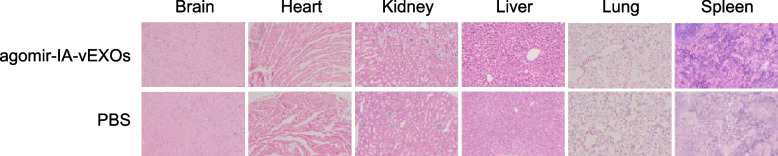
In vivo toxicity evaluation of administration. Histopathological analysis of main organs stained with hematoxylin and eosin. Images were obtained under Nikon Ti microscope using a × 10 objective

Considering the application of agomir-IA-vEXOs in pregnant mice, toxicity is a critical parameter for any excellent delivery system. For safety purposes, we evaluated the systemic toxicity of agomir-IA-vEXOs. Compared to a PBS group, no deaths or serious body weight loss were observed in the treatment group during the study period. We further investigated potential pathological lesions induced by agomir-IA-vEXOs and involving major organs, including the heart, liver, spleen, lung, kidney, and brain. As shown in Fig. 8, these major tissues showed no evidence of histopathological abnormalities or lesions in the agomir-IA-vEXO treatment group.

## Discussion

Approximately 1.25% of childbearing women suffer from uRPL, and immune imbalances at the maternal–fetal interface have been identified as a major cause [1]. CD56^+^ dNK cell numbers increase rapidly by 70% during early pregnancy, with very low cytotoxicity and high secretion of growth-promoting cytokines. It is known that immune cells are hyper-activated and that dNK cells exhibit higher expression levels of IFN-γ in uRPL patients. High concentrations of IFN-γ have been demonstrated to influence the maternal–fetal interface imbalance by suppressing Treg and Th17 polarization, upregulating TNF-α to create a pro-inflammatory environment [39], as well as downregulating matrix metalloproteinase (MMP)-7 and MMP-9 to inhibit trophoblast invasion [40] and also exhibit embryo toxicity [41]. Furthermore, IFN-γ can interfere with the normal metabolism of vascular endothelium [42], destroy connexin in endothelial cells [43], and directly lead to endothelial apoptosis and blood vessel degeneration [44]. However, the cause of the pro-inflammatory phenotype in patients’ dNK cells remains unclear. Emerging studies have indicated that exosomes are involved in various physiological and pathological processes [20]. In our previous study, we demonstrated that a villus tissue-specific expression protein, HLA-G, is significantly downregulated in uRPL-vEXOs, leading to impaired growth factor secretion function of dNK cells [27]. Here, we revealed that exosomes isolated from uRPL villi had decreased immunosuppressive ability on dNK cells and, in particular, lacked miR-29a-3p to downregulate the expression level of IFN-γ, which acts as a pro-inflammatory cytokine and can suppress vessel formation [45]. Furthermore, we used IA-vEXOs with miR-29a-3p agomir delivered via electroporation to create a more efficient therapeutic tool. In addition, we mixed agomir-IA-vEXOs and a CFDA-approved HA-Gel to sustain the existence and release of exosomes in the uterine cavity, which safely and efficiently decreased the embryo absorption rate in the uRPL mouse model. Hence, we used engineered exosomes to treat dNK cells from patients with uRPL in vitro. We found that engineered IA-vEXOs (agomir-IA-vEXOs) could effectively downregulate IFN-γ expression levels of stimulated dNK cells. This demonstrates that engineered exosomes can play a more efficient role in reducing inflammation than physiological exosomes. This study provides insights into the pathology of dNK cells in uRPL and offers new possibilities for exploring other roles of villus-derived EVs in modulating dNK cell function and in developing therapies for uRPL.

Exosomes released from healthy IA patients participate in the immunosuppressive regulation of macrophages and T cells [19, 21]. In addition, serum exosomes isolated from patients with gestational diabetes mellitus and preeclampsia can experience dysregulated placental function [46]. Among exosome cargos, miRNAs are key players in complex immune regulation [47, 48]. To the best of our knowledge, this is the first study to compare villus-derived exosomal miRNA differences in uRPL and normal pregnancies. Previous studies regarding the role of villus-derived exosomes have used immune cells isolated from the peripheral blood or immune cell lines, which may exhibit different biological behaviors from those of DICs [21]. Instead, our experiments used primary dNK cells to provide stronger and more direct evidence of intercellular communication at the maternal–fetal interface. We found that miR-24-3p, miR-29a-3p, and miR-125a-5p were enriched in the IA-vEXO group and were significantly higher than those in the uRPL-vEXO group. These miRNAs have been shown to be associated with IFN-γ regulation and all of them were effectively transferred into dNK cells by exosomes. However, we presented evidence that only miR-29a-3p is essential for the inhibition of IFN-γ expression in dNK cells by IA-vEXOs. Firstly, as found in dNK cells, transfecting agomirs of miR-29a-3p, but not agomirs of miR-24-3p or miR-125a-5p, inhibited IFN-γ expression levels, and the expression level of miR-29a-3p in exosomes from individual patient’s villi significantly correlated with their ability to inhibit IFN-γ expression. Secondly, the inhibitory effect of exosomes on dNK cells was reversed after electroporation of the miR-29a-3p antagonist, indicating a significant contribution of miR-29a-3p to the overall effects. Finally, we found that the miR-29 family was enriched in IA-vEXOs and could directly target the 3’-UTR region of *IFN-γ* mRNA at positions 475 to 481. The relative specificity was predicted using computational models and verified using a luciferase reporter-gene assay.

Exosomes are excellent delivery systems for nucleic acids and small molecule drugs [49, 50]. Liang et al. engineered exosomes containing miR-21 inhibitors and chemotherapeutics to reverse drug resistance in colon cancer [51]. Our results showed that conditioned medium of uRPL-dNK cells (mimicking the in vivo environment) could reduce the proliferation of HUVECs, which improved after treatment with engineered exosomes. Conditioned media tube formation was also significantly enhanced in the treatment group, indicating that agomir-IA-vEXOs have a stronger effect on IFN-γ inhibition.

The abortion-prone murine model CBA/J♀ × DBA/2♂ shares characteristics similar to those of human uRPL, such as high expression of proinflammatory cytokines and normal karyotype. Evidence from the model indicates that abortion is related to systemic maternal immune inflammation and the overactivation of NK and T cells at the maternal–fetal interface [38]. Therefore, we selected this model for further therapeutic exploration. For potential clinical applications, a first consideration is the method of exosome administration, as invasive procedures are not prioritized for pregnant women. We found that intravenously injected exosomes were not enriched in the uterus (Fig. 6B, C), but could play a role in reducing inflammation in peripheral blood, as reported by Bai et al. [21]. In addition, the residence time of exosomes in the uterine cavity was < 6 h if PBS was used as the exosome solvent for uterine intracavitary injection. Therefore, we used HA-Gel, a clinically approved tool, as a carrier for exosomes. We found that this mode of delivery resulted in long-lasting, sustained release, and did not cause tissue inflammation. Therefore, a combination of tail vein injections of agomir-IA-vEXOs and intrauterine injections of agomir-IA-vEXOs-HA-Gel was employed. As anticipated, miscarriage rates in the mice were significantly decreased, and IFN-γ concentrations in peripheral blood and decidua were concomitantly decreased. These in vivo findings further support the potential application of engineered exosomes for uRPL treatment.

## Conclusion

In conclusion, the results obtained in this study provide important new insights in that vEXOs derived from villi have a key role within the establishment of maternal immune tolerance by downregulation production of IFN-γ by dNK cells. Our study offers a novel strategy for delivering therapeutic nucleic acids via exosomes to prevent immune-related uRPLs.

### Supplementary Information

**Additional file 1: Figure S1.** Verification of transfection efficiency of miRNA and characterization of electroporated cel-miR-39-IA-vEXOs. (A) qRT-PCR assay demonstrated the efficiency of agomir and antagomir transfection. *n* = 3 biological replicates of a representative experiment. (B) Fluorescence microscopy images of alive dNK cells incubated with FAM labelled control miRNA (green) for 24 h (scale bar, 150 μm). (C) qRT-PCR assay demonstrated the efficiency of electroporation of cel-miR-39 transfection. *n* = 5 biological replicates of a representative experiment. (D) Representative TEM image of IA-vEXOs and cel-miR-39-IA-vEXOs. Bar, 100 nm. ^*^P < 0.05 vs. Blank + lipo reagent (A). Ordinary one-way ANOVA (A).

**Additional file 2: Table S1.** Clinical information of all donors. **Table S2.** Characteristics of all donors. **Table S3.** Clinical information of villi donors for miRNAomic sequencing. **Table S4.** Clinical information of villi donors for spearman’s correlation analysis. **Table S5.** Sequence of primers and RNA oligo.

## Data Availability

No datasets were generated or analysed during the current study.

## Abbreviations

Th2: T helper 2 cell
RPL: Recurrent pregnancy loss
uRPL: Unexplained RPL
dNK: Decidual natural killer cell
pNK: Peripheral natural killer cell
VEGF: Vascular endothelial growth factor
IFN-γ: Interferon-gamma
EVs: Extracellular vesicles
vEXOs: Villi derived exosomes
HA-GEL: Hyaluronic acid gel
IA: Induced abortion
qRT-PCR: Quantitative real-time PCR
Huvec: Human umbilical vein endothelial cells

## References

[1] Dimitriadis E, Menkhorst E, Saito S, Kutteh WH, Brosens JJ (2020). Recurrent pregnancy loss. Nat Rev Dis Primers.

[2] Bender Atik R, Christiansen OB, Elson J, Kolte AM, Lewis S, Middeldorp S, Nelen W, Peramo B, Quenby S, Vermeulen N, Goddijn M (2018). ESHRE guideline: recurrent pregnancy loss. Hum Reprod Open.

[3] Ammon Avalos L, Galindo C, Li DK (2012). A systematic review to calculate background miscarriage rates using life table analysis. Birth Defects Res A Clin Mol Teratol.

[4] Liu Y, Gao S, Zhao Y, Wang H, Pan Q, Shao Q (2021). Decidual natural killer cells: a good nanny at the maternal-fetal interface during early pregnancy. Front Immunol.

[5] Wallace AE, Fraser R, Cartwright JE (2012). Extravillous trophoblast and decidual natural killer cells: a remodelling partnership. Hum Reprod Update.

[6] Yang X, Tian Y, Zheng L, Luu T, Kwak-Kim J (2022). The update immune-regulatory role of pro- and anti-inflammatory cytokines in recurrent pregnancy losses. Int J Mol Sci.

[7] Zhang X, Wei H (2021). Role of decidual natural killer cells in human pregnancy and related pregnancy complications. Front Immunol.

[8] Qin D, Xu H, Chen Z, Deng X, Jiang S, Zhang X, Bao S (2022). The peripheral and decidual immune cell profiles in women with recurrent pregnancy loss. Front Immunol.

[9] Gamliel M, Goldman-Wohl D, Isaacson B, Gur C, Stein N, Yamin R, Berger M, Grunewald M, Keshet E, Rais Y (2018). Trained memory of human uterine NK cells enhances their function in subsequent pregnancies. Immunity.

[10] Zhou Y, Fu B, Xu X, Zhang J, Tong X, Wang Y, Dong Z, Zhang X, Shen N, Zhai Y (2020). PBX1 expression in uterine natural killer cells drives fetal growth. Sci Transl Med.

[11] Wang F, Jia W, Fan M, Shao X, Li Z, Liu Y, Ma Y, Li YX, Li R, Tu Q, Wang YL (2021). Single-cell immune landscape of human recurrent miscarriage. Genom Proteom Bioinform.

[12] Guo C, Cai P, Jin L, Sha Q, Yu Q, Zhang W, Jiang C, Liu Q, Zong D, Li K (2021). Single-cell profiling of the human decidual immune microenvironment in patients with recurrent pregnancy loss. Cell Discov.

[13] Comba C, Bastu E, Dural O, Yasa C, Keskin G, Ozsurmeli M, Buyru F, Serdaroglu H (2015). Role of inflammatory mediators in patients with recurrent pregnancy loss. Fertil Steril.

[14] Wu Z, Wang M, Liang G, Jin P, Wang P, Xu Y, Qian Y, Jiang X, Qian J, Dong M (2021). Pro-inflammatory signature in decidua of recurrent pregnancy loss regardless of embryonic chromosomal abnormalities. Front Immunol.

[15] Takeyama R, Fukui A, Mai C, Yamamoto M, Saeki S, Yamaya A, Shibahara H (2021). Co-expression of NKp46 with activating or inhibitory receptors on, and cytokine production by, uterine endometrial NK cells in recurrent pregnancy loss. J Reprod Immunol.

[16] Yang SL, Tan HX, Niu TT, Li DJ, Wang HY, Li MQ (2021). Kynurenine promotes the cytotoxicity of NK cells through aryl hydrocarbon receptor in early pregnancy. J Reprod Immunol.

[17] Liu J, Dong P, Jia N, Wen X, Luo L, Wang S, Li J (2022). The expression of intracellular cytokines of decidual natural killer cells in unexplained recurrent pregnancy loss. J Matern Fetal Neonatal Med.

[18] Von Woon E, Greer O, Shah N, Nikolaou D, Johnson M, Male V (2022). Number and function of uterine natural killer cells in recurrent miscarriage and implantation failure: a systematic review and meta-analysis. Hum Reprod Update.

[19] Gurunathan S, Kang MH, Song H, Kim NH, Kim JH (2022). The role of extracellular vesicles in animal reproduction and diseases. J Anim Sci Biotechnol.

[20] Kalluri R, LeBleu VS (2020). The biology, function, and biomedical applications of exosomes. Science.

[21] Bai K, Lee CL, Liu X, Li J, Cao D, Zhang L, Hu D, Li H, Hou Y, Xu Y (2022). Human placental exosomes induce maternal systemic immune tolerance by reprogramming circulating monocytes. J Nanobiotechnology.

[22] Mincheva-Nilsson L (2021). Immunosuppressive protein signatures carried by syncytiotrophoblast-derived exosomes and their role in human pregnancy. Front Immunol.

[23] Kim DH, Kim H, Choi YJ, Kim SY, Lee JE, Sung KJ, Sung YH, Pack CG, Jung MK, Han B (2019). Exosomal PD-L1 promotes tumor growth through immune escape in non-small cell lung cancer. Exp Mol Med.

[24] Gong Z, Li Q, Shi J, Liu ET, Shultz LD, Ren G (2022). Lipid-laden lung mesenchymal cells foster breast cancer metastasis via metabolic reprogramming of tumor cells and natural killer cells. Cell Metab.

[25] Tian Y, Gong M, Hu Y, Liu H, Zhang W, Zhang M, Hu X, Aubert D, Zhu S, Wu L, Yan X (2020). Quality and efficiency assessment of six extracellular vesicle isolation methods by nano-flow cytometry. J Extracell Vesicles.

[26] Tian Y, Ma L, Gong M, Su G, Zhu S, Zhang W, Wang S, Li Z, Chen C, Li L (2018). Protein profiling and sizing of extracellular vesicles from colorectal cancer patients via flow cytometry. ACS Nano.

[27] Fang Z, Mao J, Chen S, Dong J, Wang X (2022). Villi exosomes deliver HLA-G to enhance the expression of osteoglycin and pleiotrophin in decidual NK cells. Xi Bao Yu Fen Zi Mian Yi Xue Za Zhi.

[28] Zeng Y, Yao X, Liu X, He X, Li L, Liu X, Yan Z, Wu J, Fu BM (2019). Anti-angiogenesis triggers exosomes release from endothelial cells to promote tumor vasculogenesis. J Extracell Vesicles.

[29] Gentile MT, Pastorino O, Bifulco M, Colucci-D'Amato L (2019). HUVEC tube-formation assay to evaluate the impact of natural products on angiogenesis. J Vis Exp.

[30] Clément T, Salone V, Rederstorff M (2015). Dual luciferase gene reporter assays to study miRNA function. Methods Mol Biol.

[31] Agarwal V, Bell GW, Nam JW, Bartel DP (2015). Predicting effective microRNA target sites in mammalian mRNAs. Elife.

[32] Théry C, Witwer KW, Aikawa E, Alcaraz MJ, Anderson JD, Andriantsitohaina R, Antoniou A, Arab T, Archer F, Atkin-Smith GK (2018). Minimal information for studies of extracellular vesicles 2018 (MISEV2018): a position statement of the International Society for Extracellular Vesicles and update of the MISEV2014 guidelines. J Extracell Vesicles.

[33] Ferreira LMR, Meissner TB, Tilburgs T, Strominger JL (2017). HLA-G: At the Interface of Maternal-Fetal Tolerance. Trends Immunol.

[34] Buzas EI. The roles of extracellular vesicles in the immune system. Nat Rev Immunol. 2023;23:236–250. 10.1038/s41577-022-00763-8 PMC9361922 35927511

[35] Abdi K, Laky K, Abshari M, Hill EM, Lantz L, Singh NJ, Long EO (2022). Dendritic cells trigger IFN-γ secretion by NK cells independent of IL-12 and IL-18. Eur J Immunol.

[36] Song J, Song B, Yuan L, Yang G (2022). Multiplexed strategies toward clinical translation of extracellular vesicles. Theranostics.

[37] Jiang L, Fei H, Jin X, Liu X, Yang C, Li C, Chen J, Yang A, Zhu J, Wang H (2021). Extracellular vesicle-mediated secretion of HLA-E by trophoblasts maintains pregnancy by regulating the metabolism of decidual NK Cells. Int J Biol Sci.

[38] Bonney EA, Brown SA (2014). To drive or be driven: the path of a mouse model of recurrent pregnancy loss. Reproduction.

[39] Piccinni MP, Raghupathy R, Saito S, Szekeres-Bartho J (2021). Cytokines, hormones and cellular regulatory mechanisms favoring successful reproduction. Front Immunol.

[40] He Y, Sun Q (2018). IFN-γ induces upregulation of TNF-α, downregulation of MMP-2 and MMP-9 expressions in abortion rat. Eur Rev Med Pharmacol Sci.

[41] Robertson SA, Chin PY, Femia JG, Brown HM (2018). Embryotoxic cytokines-Potential roles in embryo loss and fetal programming. J Reprod Immunol.

[42] Lee LY, Oldham WM, He H, Wang R, Mulhern R, Handy DE, Loscalzo J (2021). Interferon-γ impairs human coronary artery endothelial glucose metabolism by tryptophan catabolism and activates fatty acid oxidation. Circulation.

[43] Langer V, Vivi E, Regensburger D, Winkler TH, Waldner MJ, Rath T, Schmid B, Skottke L, Lee S, Jeon NL (2019). IFN-γ drives inflammatory bowel disease pathogenesis through VE-cadherin-directed vascular barrier disruption. J Clin Invest.

[44] Kammertoens T, Friese C, Arina A, Idel C, Briesemeister D, Rothe M, Ivanov A, Szymborska A, Patone G, Kunz S (2017). Tumour ischaemia by interferon-γ resembles physiological blood vessel regression. Nature.

[45] Weyand CM, Goronzy JJ (2013). Immune mechanisms in medium and large-vessel vasculitis. Nat Rev Rheumatol.

[46] Wang M, Zheng L, Ma S, Lin R, Li J, Yang S (2023). Biogenesis and function of exosome lncRNAs and their role in female pathological pregnancy. Front Endocrinol (Lausanne).

[47] Rezaie J, Etemadi T, Feghhi M (2022). The distinct roles of exosomes in innate immune responses and therapeutic applications in cancer. Eur J Pharmacol.

[48] Xu Z, Chen Y, Ma L, Chen Y, Liu J, Guo Y, Yu T, Zhang L, Zhu L, Shu Y (2022). Role of exosomal non-coding RNAs from tumor cells and tumor-associated macrophages in the tumor microenvironment. Mol Ther.

[49] Liang Y, Duan L, Lu J, Xia J (2021). Engineering exosomes for targeted drug delivery. Theranostics.

[50] Xu H, Jia S, Xu H (2019). Potential therapeutic applications of exosomes in different autoimmune diseases. Clin Immunol.

[51] Liang G, Zhu Y, Ali DJ, Tian T, Xu H, Si K, Sun B, Chen B, Xiao Z (2020). Engineered exosomes for targeted co-delivery of miR-21 inhibitor and chemotherapeutics to reverse drug resistance in colon cancer. J Nanobiotechnology.

