# Supplementary figures and images for: Increased levels of villus-derived exosomal miR-29a-3p in normal pregnancy than uRPL patients suppresses decidual NK cell production of interferon-γ and exerts a therapeutic effect in abortion-prone mice
Source: Cell Commun Signal. 2024 Apr 16;22:230. doi: 10.1186/s12964-024-01610-0 (PMC11022359; doi:10.1186/s12964-024-01610-0)

A

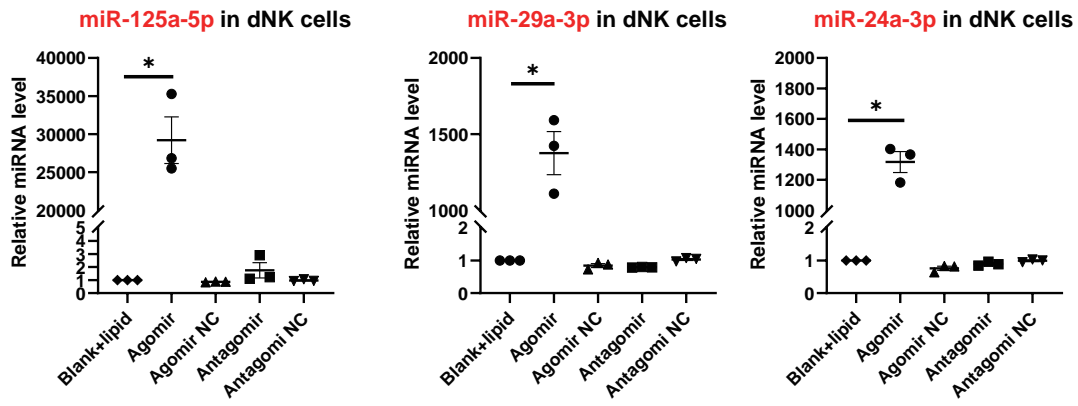

B

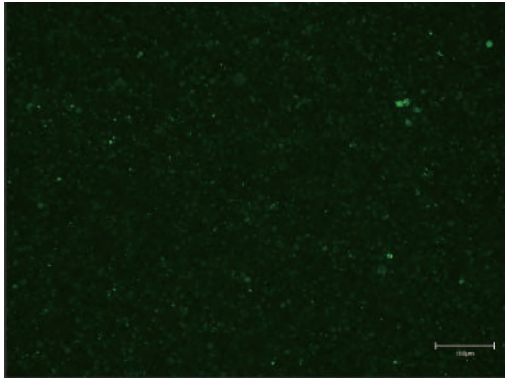

C

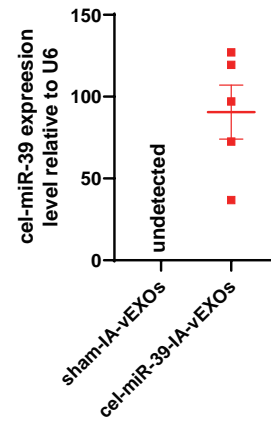

D

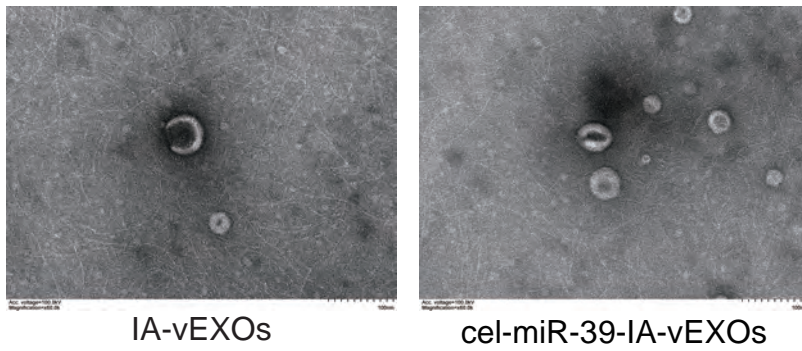

Supplement: Supplementary file 1 — Additional file 1: Figure S1. Verification of transfection efficiency of miRNA and characterization of electroporated cel-miR-39-IA-vEXOs. (A) qRT-PCR assay demonstrated the efficiency of agomir and antagomir transfection. n = 3 biological replicates of a representative experiment. (B) Fluorescence microscopy images of alive dNK cells incubated with FAM labelled control miRNA (green) for 24 h (scale bar, 150 μm). (C) qRT-PCR assay demonstrated the efficiency of electroporation of cel-miR-39 transfection. n = 5 biological replicates of a representative experiment. (D) Representative TEM image of IA-vEXOs and cel-miR-39-IA-vEXOs. Bar, 100 nm. *P < 0.05 vs. Blank + lipo reagent (A). Ordinary one-way ANOVA (A). [file 12964_2024_1610_MOESM1_ESM.pdf]
