# Supplementary material for: Increased levels of villus-derived exosomal miR-29a-3p in normal pregnancy than uRPL patients suppresses decidual NK cell production of interferon-γ and exerts a therapeutic effect in abortion-prone mice
Source: Cell Commun Signal. 2024 Apr 16;22:230. doi: 10.1186/s12964-024-01610-0 (PMC11022359; doi:10.1186/s12964-024-01610-0)
Supplement: Supplementary file 2 — Additional file 2: Table S1. Clinical information of all donors. Table S2. Characteristics of all donors. Table S3. Clinical information of villi donors for miRNAomic sequencing. Table S4. Clinical information of villi donors for spearman’s correlation analysis. Table S5. Sequence of primers and RNA oligo. [file 12964_2024_1610_MOESM2_ESM.docx]

**Table S1.** Clinical information of all donors.

| Donor | Age | BMI | Gestational days | No. of pregnancy | No. of spontaneous abortion |
| --- | --- | --- | --- | --- | --- |
| IA donor 1 | 35 | 20.6 | 42 | 3 | 0 |
| IA donor 2 | 20 | 19.4 | 54 | 0 | 0 |
| IA donor 3 | 29 | 20.4 | 48 | 1 | 0 |
| IA donor 4 | 32 | 20.4 | 51 | 3 | 0 |
| IA donor 5 | 33 | 22.5 | 44 | 2 | 0 |
| IA donor 6 | 25 | 19.3 | 53 | 1 | 0 |
| IA donor 7 | 30 | 21.1 | 50 | 2 | 0 |
| IA donor 8 | 30 | 22.5 | 47 | 1 | 0 |
| IA donor 9 | 20 | 18.6 | 48 | 1 | 0 |
| IA donor 10 | 20 | 18.3 | 42 | 1 | 0 |
| IA donor 11 | 30 | 21.4 | 51 | 2 | 0 |
| IA donor 12 | 33 | 21.7 | 53 | 1 | 0 |
| IA donor 13 | 24 | 20.9 | 56 | 1 | 0 |
| IA donor 14 | 19 | 19.4 | 44 | 1 | 0 |
| IA donor 15 | 29 | 21.4 | 50 | 2 | 0 |
| IA donor 16 | 28 | 23.1 | 47 | 1 | 0 |
| IA donor 17 | 24 | 21.1 | 50 | 1 | 0 |
| IA donor 18 | 23 | 22.5 | 50 | 1 | 0 |
| IA donor 19 | 22 | 21 | 45 | 1 | 0 |
| IA donor 20 | 26 | 17.4 | 45 | 3 | 0 |
| IA donor 21 | 23 | 20.1 | 50 | 1 | 0 |
| IA donor 22 | 32 | 18.4 | 46 | 1 | 0 |
| IA donor 23 | 19 | 20.5 | 52 | 1 | 0 |
| IA donor 24 | 27 | 20.4 | 45 | 1 | 0 |
| IA donor 25 | 24 | 21.9 | 48 | 1 | 0 |
| IA donor 26 | 21 | 17.4 | 50 | 0 | 0 |
| IA donor 27 | 38 | 25.6 | 42 | 2 | 0 |
| IA donor 28 | 25 | 19.3 | 44 | 3 | 0 |
| IA donor 29 | 23 | 20.7 | 46 | 4 | 0 |
| IA donor 30 | 34 | 21.6 | 47 | 0 | 0 |
| IA donor 31 | 37 | 23.8 | 47 | 2 | 0 |
| IA donor 32 | 20 | 18.2 | 48 | 1 | 0 |
| IA donor 33 | 33 | 21.5 | 53 | 1 | 0 |
| IA donor 34 | 31 | 19.6 | 55 | 2 | 0 |
| IA donor 35 | 25 | 20.4 | 42 | 0 | 0 |
| IA donor 36 | 35 | 21.5 | 51 | 2 | 0 |
| IA donor 37 | 28 | 21.4 | 46 | 1 | 0 |
| IA donor 38 | 26 | 19.8 | 48 | 1 | 0 |
| IA donor 39 | 20 | 20.8 | 47 | 0 | 0 |
| IA donor 40 | 34 | 22.8 | 49 | 2 | 0 |
| IA donor 41 | 30 | 20.7 | 53 | 1 | 0 |
| IA donor 42 | 31 | 21.2 | 53 | 1 | 0 |
| IA donor 43 | 34 | 21.3 | 42 | 3 | 0 |
| IA donor 44 | 26 | 18.9 | 52 | 1 | 0 |
| IA donor 45 | 25 | 17.2 | 42 | 1 | 0 |
| IA donor 46 | 25 | 20.3 | 45 | 0 | 0 |
| IA donor 47 | 33 | 23.6 | 49 | 2 | 0 |
| IA donor 48 | 33 | 22.1 | 49 | 3 | 0 |
| IA donor 49 | 33 | 20.4 | 47 | 1 | 0 |
| IA donor 50 | 33 | 19.6 | 54 | 1 | 0 |
| IA donor 51 | 36 | 20.2 | 46 | 4 | 0 |
| IA donor 52 | 33 | 22.1 | 50 | 3 | 0 |
| IA donor 53 | 27 | 21.5 | 48 | 2 | 0 |
| IA donor 54 | 21 | 22.7 | 55 | 1 | 0 |
| IA donor 55 | 30 | 20.6 | 52 | 1 | 0 |
| IA donor 56 | 28 | 23.2 | 45 | 1 | 0 |
| IA donor 57 | 31 | 22.9 | 48 | 2 | 0 |
| IA donor 58 | 32 | 21.4 | 46 | 1 | 0 |
| IA donor 59 | 35 | 22.8 | 56 | 2 | 0 |
| IA donor 60 | 33 | 19.3 | 42 | 3 | 0 |
| IA donor 61 | 31 | 20.2 | 48 | 1 | 0 |
| IA donor 62 | 25 | 21.7 | 51 | 1 | 0 |
| IA donor 63 | 25 | 21.4 | 56 | 1 | 0 |
| IA donor 64 | 31 | 23.4 | 48 | 2 | 0 |
| IA donor 65 | 22 | 20.5 | 46 | 1 | 0 |
| IA donor 66 | 24 | 21.6 | 46 | 1 | 0 |
| IA donor 67 | 22 | 20.9 | 53 | 1 | 0 |
| IA donor 68 | 23 | 21.8 | 48 | 1 | 0 |
| IA donor 69 | 19 | 19.5 | 55 | 0 | 0 |
| IA donor 70 | 30 | 21.4 | 47 | 1 | 0 |
| IA donor 71 | 30 | 20.4 | 48 | 1 | 0 |
| IA donor 72 | 26 | 20.4 | 48 | 1 | 0 |
| IA donor 73 | 30 | 22.3 | 47 | 1 | 0 |
| IA donor 74 | 18 | 17.5 | 46 | 0 | 0 |
| IA donor 75 | 34 | 24.2 | 44 | 1 | 0 |
| IA donor 76 | 18 | 19.4 | 49 | 0 | 0 |
| IA donor 77 | 29 | 19.6 | 43 | 2 | 0 |
| IA donor 78 | 19 | 18.4 | 45 | 0 | 0 |
| IA donor 79 | 29 | 21.3 | 44 | 3 | 0 |
| IA donor 80 | 32 | 21.1 | 49 | 3 | 0 |
| IA donor 81 | 28 | 21.4 | 49 | 0 | 0 |
| IA donor 82 | 33 | 25.5 | 52 | 1 | 0 |
| IA donor 83 | 29 | 20.3 | 51 | 1 | 0 |
| IA donor 84 | 31 | 21.2 | 52 | 2 | 0 |
| IA donor 85 | 22 | 20.5 | 49 | 0 | 0 |
| uRPL donor 1 | 34 | 34 | 56 | 4 | 4 |
| uRPL donor 2 | 25 | 20.9 | 54 | 2 | 2 |
| uRPL donor 3 | 27 | 19.6 | 49 | 2 | 2 |
| uRPL donor 4 | 28 | 23.6 | 49 | 2 | 2 |
| uRPL donor 5 | 33 | 22.4 | 50 | 2 | 2 |
| uRPL donor 6 | 38 | 21.4 | 54 | 2 | 2 |
| uRPL donor 7 | 31 | 20.8 | 52 | 3 | 3 |
| uRPL donor 8 | 26 | 21.4 | 51 | 2 | 2 |
| uRPL donor 9 | 35 | 22.7 | 56 | 2 | 2 |
| uRPL donor 10 | 36 | 22.4 | 55 | 7 | 4 |
| uRPL donor 11 | 26 | 20.1 | 49 | 2 | 2 |
| uRPL donor 12 | 28 | 20.1 | 53 | 2 | 2 |
| uRPL donor 13 | 30 | 22.1 | 53 | 2 | 2 |
| uRPL donor 14 | 23 | 21.3 | 54 | 2 | 2 |
| uRPL donor 15 | 35 | 18.6 | 53 | 3 | 3 |
| uRPL donor 16 | 28 | 21.7 | 50 | 3 | 3 |
| uRPL donor 17 | 26 | 24.2 | 50 | 2 | 2 |
| uRPL donor 18 | 30 | 23.3 | 50 | 2 | 2 |
| uRPL donor 19 | 28 | 18.6 | 54 | 2 | 2 |
| uRPL donor 20 | 30 | 21.5 | 50 | 2 | 2 |
| uRPL donor 21 | 32 | 20.4 | 54 | 2 | 2 |
| uRPL donor 22 | 25 | 21.3 | 50 | 2 | 2 |
| uRPL donor 23 | 27 | 22.5 | 53 | 2 | 2 |
| uRPL donor 24 | 32 | 21.9 | 55 | 3 | 3 |
| uRPL donor 25 | 37 | 22.4 | 50 | 2 | 2 |
| uRPL donor 26 | 27 | 20.4 | 55 | 3 | 3 |
| uRPL donor 27 | 23 | 20.3 | 50 | 3 | 3 |
| uRPL donor 28 | 33 | 21.4 | 54 | 3 | 3 |
| uRPL donor 29 | 26 | 21.9 | 54 | 2 | 2 |
| uRPL donor 30 | 33 | 22.3 | 50 | 2 | 2 |
| uRPL donor 31 | 38 | 20.7 | 56 | 2 | 2 |
| uRPL donor 32 | 27 | 22.8 | 53 | 2 | 2 |
| uRPL donor 33 | 30 | 21.7 | 50 | 2 | 2 |
| uRPL donor 34 | 35 | 22.4 | 50 | 2 | 2 |
| uRPL donor 35 | 30 | 21.9 | 50 | 2 | 2 |
| uRPL donor 36 | 23 | 21.7 | 50 | 2 | 2 |
| uRPL donor 37 | 27 | 22.4 | 54 | 2 | 2 |
| uRPL donor 38 | 32 | 22.1 | 55 | 2 | 2 |
| uRPL donor 39 | 37 | 21.4 | 50 | 2 | 2 |

**Table S2.** Characteristics of all donors.

| Donor | IA (n=85) | uRPL (n=39) |
| --- | --- | --- |
| Age (median ± IQR) | 29.00(23.50-32.00) | 30.00(27.00-33.00) |
| BMI (median ± IQR) | 20.90(19.95-21.70) | 21.70(20.80-22.40) |
| Gestational days (median ± IQR) | 48.00(46.00-51.00) | 53.00(50.00-54.00) |
| No. of pregnancy [mean (range)] | 1.35(4.00) | 2.36(5.00) |
| No. of spontaneous abortion [mean (range)] | 0.00(0.00) | 2.28(2.00) |

**Table S3.** Clinical information of villi donors for miRNAomic sequencing

| Donor | Age | BMI | Gestational days | No. of pregnancy | No. of spontaneous abortion |
| --- | --- | --- | --- | --- | --- |
| IA donor 1 | 31 | 21.2 | 52 | 2 | 0 |
| IA donor 2 | 30 | 20.7 | 53 | 1 | 0 |
| IA donor 3 | 33 | 21.7 | 53 | 1 | 0 |
| uRPL donor 1 | 31 | 20.8 | 52 | 3 | 3 |
| uRPL donor 2 | 30 | 22.1 | 53 | 2 | 2 |
| uRPL donor 3 | 35 | 18.6 | 53 | 3 | 3 |
|  |  |  |  |  |  |
| P-value^a^ | >0.999 | >0.999 | >0.999 | 0.200 | 0.100 |

a: P-value was calculated by two-tailed Mann Whitney test for non-normally distributed data.

**Table S4.** Clinical information of villi donors for spearman’s correlation analysis

| Donor | Age | BMI | Gestational days | No. of pregnancy | No. of spontaneous abortion |
| --- | --- | --- | --- | --- | --- |
| IA donor 1 | 22 | 20.5 | 49 | 0 | 0 |
| IA donor 2 | 28 | 21.4 | 49 | 0 | 0 |
| IA donor 3 | 32 | 21.1 | 49 | 3 | 0 |
| IA donor 4 | 18 | 19.4 | 49 | 0 | 0 |
| IA donor 5 | 33 | 23.6 | 49 | 2 | 0 |
| IA donor 6 | 33 | 22.1 | 49 | 3 | 0 |
| IA donor 7 | 33 | 22.1 | 50 | 3 | 0 |
| IA donor 8 | 26 | 18.9 | 52 | 1 | 0 |
| IA donor 9 | 31 | 21.2 | 53 | 1 | 0 |
| IA donor 10 | 33 | 21.5 | 53 | 1 | 0 |
| uRPL donor 1 | 28 | 23.6 | 49 | 2 | 2 |
| uRPL donor 2 | 27 | 19.6 | 49 | 2 | 2 |
| uRPL donor 3 | 26 | 20.1 | 49 | 2 | 2 |
| uRPL donor 4 | 23 | 20.3 | 50 | 2 | 2 |
| uRPL donor 5 | 30 | 21.9 | 50 | 2 | 2 |
| uRPL donor 6 | 23 | 21.7 | 50 | 2 | 2 |
| uRPL donor 7 | 31 | 20.8 | 52 | 3 | 3 |
| uRPL donor 8 | 30 | 22.1 | 53 | 2 | 2 |
| uRPL donor 9 | 35 | 18.6 | 53 | 3 | 3 |
| uRPL donor 10 | 27 | 22.5 | 53 | 2 | 2 |
|  |  |  |  |  |  |
| P-value^a^ | 0.404 | 0.989 | 0.371 | 0.125 | <0.001 |

a: P-value was calculated by two-tailed Mann Whitney test for non-normally distributed data.

**Table S5.** Sequence of primers and RNA oligo.

| **Primer（human）** | **Sequence（5’-3’）** |
| --- | --- |
| hsa-miR-29a-3p | CGCTAGCACCATCTGAAATCGGTTA |
| hsa-miR-24a-3p | TGGCTCAGTTCAGCAGGAACAG |
| hsa-miR-125a-5p | TCCCTGAGACCCTTTAACCTGTGA |
| hsa-miR-29b-3p | TTGTGACTAAAGTTTACCACGAT |
| hsa-miR-29c-3p | ATTGGCTAAAGTTTACCACGAT |
| cel-miR-39-3p | TCACCGGGTGTAAATCAGCTTG |
| U6-Forward | GGAACGATACAGAGAAGATTAGC |
| U6-Reverse | TGGAACGCTTCACGAATTTGCG |
| *Gapdh*-Forward | GGAGCGAGATCCCTCCAAAAT |
| *Gapdh*-Reverse | GGCTGTTGTCATACTTCTCATGG |
| *Ifn-γ*-Forward | TTTTGCCAAGGAGTGCTAAAGA |
| *Ifn-γ*-Reverse | TCGCTTCCCTGTTTTAGCTGC |

| **Primer（mouse）** | **Sequence（5’-3’）** |
| --- | --- |
| *Ifn-γ*- Forward | ATGAACGCTACACACTGCATC |
| *Ifn-γ*- Reverse | CCATCCTTTTGCCAGTTCCTC |
| *Gapdh*-Forward | AGGTCGGTGTGAACGGATTTG |
| *Gapdh*-Reverse | TGTAGACCATGTAGTTGAGGTCA |

| **RNA oligo** | **Sequence（5’- 3’）** |
| --- | --- |
| hsa-miR-29a-3p agomir | UAGCACCAUCUGAAAUCGGUUA  ACCGAUUUCAGAUGGUGCUAUU |
| hsa-miR-24a-3p agomir | UGGCUCAGUUCAGCAGGAACAG  GUUCCUGCUGAACUGAGCCAUU |
| hsa-miR-125a-5p agomir | UCCCUGAGACCCUUUAACCUGUGA  ACAGGUUAAAGGGUCUCAGGGAUU |
| hsa-miR-29a-3p antagomir | UAACCGAUUUCAGAUGGUGCUA |
| hsa-miR-24a-3p antagomir | CUGUUCCUGCUGAACUGAGCCA |
| hsa-miR-125a-5p antagomir | UCACAGGUUAAAGGGUCUCAGGGA |
| Stable negative control | UUCUCCGAACGUGUCACGUTT  ACGUGACACGUUCGGAGAATT |
| Negative control FAM | UUCUCCGAACGUGUCACGUTT  ACGUGACACGUUCGGAGAATT |
| MicroRNA inhibitor N.C. | CAGUACUUUUGUGUAGUACAA |
| cel-miR-39-3p | UCACCGGGUGUAAAUCAGCUUG |
